# EGF receptor trafficking: consequences for signaling and cancer

**DOI:** 10.1016/j.tcb.2013.11.002

**Published:** 2014-01

**Authors:** Alejandra Tomas, Clare E. Futter, Emily R. Eden

**Affiliations:** University College London (UCL) Institute of Ophthalmology, London, UK

**Keywords:** epidermal growth factor receptor (EGFR), endocytosis, trafficking, ubiquitination, oncogenes, antineoplastic therapy

## Abstract

The ligand-stimulated epidermal growth factor receptor (EGFR) has been extensively studied in the analysis of molecular mechanisms regulating endocytic traffic and the role of that traffic in signal transduction. Although such studies have largely focused on mitogenic signaling and dysregulated traffic in tumorigenesis, there is growing interest in the potential role of EGFR traffic in cell survival and the consequent response to cancer therapy. Here we review recent advances in our understanding of molecular mechanisms regulating ligand-stimulated EGFR activation, internalization, and post-endocytic sorting. The role of EGFR overexpression/mutation and new modulators of EGFR traffic in cancer and the response to cancer therapeutics are also discussed. Finally, we speculate on the relationship between EGFR traffic and cell survival.

## Highlights

• EGF receptor endocytic traffic can regulate signaling and cell survival.
• Signaling from activated EGFR occurs at the endosome as well as the cell surface.
• Endocytosis can have positive and negative effects on signaling and tumorigenesis.
• EGFR traffic promoted by antineoplastic therapy is important in tumor resistance.

## Overview of EGFR signaling regulation

EGFR plays key roles in essential cellular functions including proliferation and migration. However, its aberrant activity in the pathogenesis of human cancers underlies our need to understand the complex regulation of EGFR activity and downstream signaling events. Widely considered as the prototypic receptor tyrosine kinase (RTK), EGFR endocytic traffic and regulation has been the subject of considerable scrutiny but although enormous advances have been made our understanding remains incomplete.

**Figure 1:**
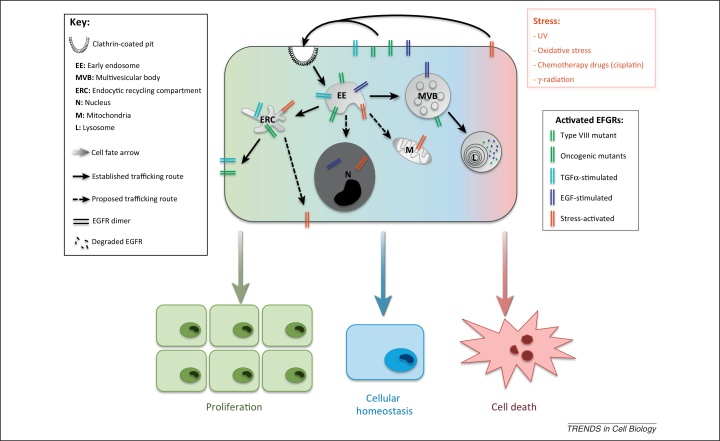
EGF receptor (EGFR) trafficking pathways and associated outcomes. Activated cell-surface EGFRs are internalized and sorted at the early endosome. The fate of the receptor has important consequences for biological cell outputs, with the recycling pathway favoring cell proliferation (depicted green), although the degradative pathway via ESCRT (endosomal sorting complex required for transport)-mediated sorting within multivesicular bodies (MVBs)/lysosomes correlates with normal cellular homeostasis (depicted in blue). Atypical trafficking pathways to the nucleus and mitochondria have also been described and are proposed to favor survival, but the transport mechanisms are not well established. Exposure to stress leads to the removal of the receptor from the cell surface, and this has been proposed to potentiate cell death (depicted in red). Conversely, stress-activated receptor might also be recycled, thereby promoting cell survival and/or proliferation.

The prevailing consensus has historically viewed endocytic transport of activated RTKs as a means of signal attenuation. This view is supported by the enhanced EGF-stimulated mitogenic signaling and proliferation in cells expressing either a non-internalizing mutant EGFR [1] or a dynamin mutant that prevents clathrin-mediated endocytosis [2]. Moreover, EGF-stimulated MAPK (mitogen-activated protein kinase) signaling was found to occur primarily at the plasma membrane, independently of dynamin activity [3]. However, a more complex picture of multifaceted, spatial, and temporal regulation is emerging. EGFR can be activated by ligand-independent mechanisms as well as by multiple ligands, often with differing signaling outcomes (Figure 1). Moreover, although the majority of EGFR signaling is believed to occur at the plasma membrane 3, 4, activated EGFR-mediated signals can continue from endosomes, suggesting distinct signaling pathways exist that actively require EGFR endocytosis 2, 5.

This review addresses the regulation of EGFR endocytic trafficking and the consequences of this regulation for the spatiotemporal control of signaling and cell fate in normal conditions, as well as in tumorigenesis and the response to cancer therapy.

## Ligand-stimulated EGFR activation

To date eight EGFR ligands have been described. Crystallography studies have revealed that ligand-mediated EGFR activation is achieved by a conformational change in the extracellular domain of the receptor upon ligand binding, resulting in receptor dimerization and internalization [6]. Unliganded EGFR can also be internalized but at a 10-fold slower rate than EGF-stimulated receptor [7], but the dimerization and activation states of these receptors are unclear. Crucial to RTK activation is the formation of an asymmetric dimer of kinase domains [8]. Recent data have implicated cytohesins, guanine nucleotide exchange factors for ADP-ribosylation factors, in this process [9], and cytohesin-mediated conformational modification of EGF-bound receptor dimers from the cytoplasmic side is reported to increase RTK activity. The EGFR has also been shown to fluctuate between monomer and dimer states, even in the absence of ligand, but ligand binding was found to promote dimerization and be required for signaling [10]. Consistent with this, it has recently been proposed that EGFR autoinhibition in the absence of EGF exists when the receptor is on the plasma membrane, and is relieved by interaction between transmembrane helices on ligand binding or under conditions of very high receptor expression [11]. Other factors implicated in the regulation of EGFR activation include association with flotillins at the plasma membrane [12] and interaction with Ca^2+^/calmodulin complexes via the calmodulin-binding domain at the cytosolic juxtamembrane region of the EGFR [13]. Active EGFR dimers undergo autophosphorylation of tyrosine residues in the cytoplasmic tail of the receptor. Consequently, phosphotyrosine-binding proteins are recruited, activating multiple signal transduction pathways, including the MAPK signaling cascade, the phosphoinositide-3-kinase (PI3K) pathway, which recruits Akt/PKB to the plasma membrane, and the phospholipase Cγ pathway, which directly interacts with EGFR, leading to protein kinase C (PKC) activation (reviewed in [14]).

## Ligand-stimulated EGFR endocytosis: a positive and negative regulator of signaling

Signaling from activated EGFR, including the MAPK signaling cascade as well as PI3K activation, occurs mostly from the plasma membrane 3, 4, and endocytosis is thought to initiate termination of the signal. However, internalization of activated EGFR also enables specific signaling pathways from intracellular sites, and endocytic trafficking of EGFRs is required for optimal activation of a subset of signal transducers [2].

### The clathrin adaptor protein complex AP2

Some controversy exists over the mechanisms of EGFR endocytosis. The involvement of the clathrin adaptor protein complex AP2 in EGFR endocytosis has been the subject of debate following conflicting reports of the effects of small interfering RNA (siRNA)-mediated depletion of AP2 on EGFR endocytosis 15, 16. Interaction between EGFR and the μ2 subunit of AP2 has been demonstrated (reviewed in [17]). Moreover, the AP2 β2 subunit becomes tyrosine-phosphorylated upon EGF stimulation, a process dependent on the dileucine motif in the EGFR carboxyl terminus [18]. Mutation of this motif, however, did not affect EGFR endocytosis, but targeting of the receptor for degradation was disrupted, suggesting that either the EGFR dileucine motif or AP2 β2 phosphorylation might facilitate the recruitment of downstream sorting machinery. Taken together, these studies suggest that although AP2 is not an absolute requirement of clathrin-mediated endocytosis (CME) of the EGFR its interaction with EGFR and role in recruiting other components of the endocytic machinery can facilitate endocytosis and the passage of activated EGFR through the endocytic pathway.

### Grb2 and EGFR ubiquitination

Another EGFR-binding adaptor protein is growth factor receptor-bound protein 2 (Grb2), which binds activated EGFR through its Src homology 2 (SH2) domain [19]. This interaction mediates apparently opposing effects on signaling: downregulation through internalization and ubiquitination-targeted degradation but also activation of signaling cascades through interaction of Grb2 SH3 domains with the Ras guanine exchange factor, son of sevenless homolog (SOS) [20]. Grb2 recruits the E3 ubiquitin ligase Cbl, resulting in monomeric and polymeric ubiquitination on lysine residues in the kinase domain of the EGFR [21] as well as modification with monomers of the ubiquitin-like molecule NEDD8 (neural precursor cell expressed developmentally downregulated protein 8) [22]. Interaction with the GTP-bound active form of Ras results in Raf activation and initiation of the MAPK signaling cascade as well as activation of Cdc42 and PI3K. PI3K activation is also mediated by interaction of Grb2 and Gab1 [23], and functions in a positive feedback loop, producing PI3,4,5P_3_, which targets Gab1 to the plasma membrane in response to EGF stimulation, where direct Gab1–EGFR interaction potentiates MAPK activation [24].

EGFR ubiquitination is recognized by ubiquitin-binding proteins of the clathrin coat, including the AP2-interacting proteins epsin1 and Eps15. Although ubiquitination has been reported to facilitate recruitment of activated EGFR to clathrin-coated pits and promote CME 25, 26, a receptor lacking 15 lysine residues in the kinase domain is negligibly ubiquitinated but is internalized normally [27]. Moreover, EGFR CME was largely unaffected by epsin1 or Eps15 depletion 28, 29, although impaired internalization because of epsin1 depletion was also reported [30]. The extent of the contribution of ubiquitination and ubiquitination-dependent effectors (such as epsin and Eps15) to EGFR internalization may depend on cell type and physiological conditions. A recent study, mutating 21 lysine residues, three of which were found to be acetylated, resulted in impaired internalization [1], raising the intriguing possibility that acetylation might be required for EGFR dimerization, similarly to STAT3 [31] and the prolactin receptor. Dimerization is crucial for ligand-stimulated EGFR autophosphorylation, activation, and internalization, with the exception of constitutively active mutant receptors (see ‘EGFR oncogenic mutations’, below). Interestingly the dimerization state of EGFR activated ligand-independently has not yet been established. Using multiple EGFR mutations deficient in ubiquitination, acetylation, and interaction with AP-2 and Grb2, Goh *et al.* [1] concluded that regulation of CME of the EGFR is complex, involving a combination of all of the above factors functioning both redundantly and cooperatively.

### Clathrin-independent EGFR endocytosis

CME offers a rapid internalization pathway, but slower clathrin-independent mechanisms have also been reported, and ligand concentration is thought to be important in directing receptor passage through the endocytic pathway. High EGF concentrations (20 ng/ml) were found to promote clathrin-independent endocytosis (CIE) [32] in an epsin and eps15-dependent manner [33], possibly due to saturation of the clathrin-mediated pathway. Surprisingly, this study found that CME promoted EGFR recycling with prolonged signaling as a consequence. Consistent with this, Dynamin2 mutants were found to reduce CIE of EGFR without affecting uptake of transferrin receptor, resulting in reduced EGFR degradation [34]. Recently a Grb2/Cbl-dependent ubiquitin threshold was described that correlates with EGFR CIE and downregulation of signal transduction, although exactly how ubiquitination and CIE are coupled remains unclear [35]. It should be noted that CIE of EGFR remains controversial because, in separate studies, clathrin depletion was found to inhibit EGFR endocytosis even at high ligand concentrations, with the disparity being attributed to the relative efficiencies of clathrin depletion 36, 37. The type of activating ligand itself is also important. Receptor activation by different ligands, EGF or transforming growth factor (TGF-α), resulted in CME alone whereas the most potent activators of EGFR, heparin-binding EGF-like growth factor (HB-EGF) and betacellulin (BTC), stimulated both clathrin-mediated and clathrin-independent mechanisms [37]. The authors suggested that, although this could be due to more efficient recruitment of residual clathrin following this potent activation in clathrin-depleted cells, an alternative as yet unidentified internalization pathway might be employed under these conditions. Proposed mechanisms of CIE include uptake via caveolae [38] or via macropinocytosis, as observed in response to EGF stimulation in A-431 cells [39], or in response to antibody binding in porcine aortic endothelial cells [40]. Thus, a growing body of evidence is emerging in support of a clathrin-independent, dynamin2-dependent mechanism of internalization of EGFR that may operate under particular physiological conditions, perhaps involving saturation of the clathrin machinery or a cellular response to potent RTK activation (by ligand type or concentration), resulting in increased traffic along the degradative pathway to maintain signal homeostasis.

## Post-endocytic EGFR sorting

Two major destinations exist for EGFR trafficking from early endosomes: recycling to the cell surface or lysosomal degradation. A delicate equilibrium between these two pathways balances continued signaling both from endosomes and recycled EGFR at the cell surface against signal attenuation in the degradative pathway. Furthermore, subcompartmentalization of signaling has been shown to occur within the endocytic pathway. A subpopulation of early endosomes has been described that are positive for the Rab5 effector APPL1, which was found to regulate AKT activity from early endosomes [41]. Moreover, the Regulator complex, comprising LAMTOR-1, -2, and -3, which interacts with MEK1/ERK1, regulates continued EGF-dependent MAPK signaling from late endosomes and lysosomes, and promotes *in vivo* proliferation [42].

### Receptor ubiquitination and ESCRT *(endosomal sorting complex required for transport)*-mediated sorting within multivesicular bodies (MVBs)

As well as being recognized by components of the endocytic machinery at the plasma membrane, receptor ubiquitination is crucial to sorting activated EGFR at the endosome ([43] for review). The ESCRT machinery sorts ubiquitinated receptor onto the intraluminal vesicles (ILVs) of maturing endosomes, thereby physically removing the active kinase domain from cytosolic substrates as well as targeting the ubiquitinated receptor for lysosomal degradation. Ubiquitinated receptors are recognized by several ubiquitin interacting motif (UIM)-containing proteins including the Rab5 exchange factor, Rabex-5 [44], the ESCRT-0 component, hepatocyte growth-factor regulated tyrosine kinase substrate (Hrs), and the ESCRT-I component, tumor susceptibility gene 101 (Tsg101). Indeed, EGFR ubiquitination is required for its interaction with Hrs and efficient lysosomal targeting [45]. Hrs interaction with the ESCRT-I component, Tsg101, promotes recruitment of subsequent ESCRT complexes, with ESCRT-III-dependent scission completing ILV biogenesis. Before sorting onto ILVs, the ubiquitin is removed by deubiquitinating enzymes (DUBs), and recycled to maintain the free ubiquitin pool. The DUBs AMSH (associated molecule with the SH3 domain of STAM) [46] and UBPY (ubiquitin isopeptidase Y/ubiquitin-specific protease 8) [47], bind both the ESCRT-0 component STAM (signal transducing adaptor molecule) and ESCRT-III complex members [48], and are implicated in regulating EGFR sorting onto the degradative pathway. By contrast, Cezanne-1, a DUB that is overexpressed in breast cancer, has recently been reported to prevent EGFR degradation, resulting in enhanced oncogenic signaling [49]. As an additional level of EGF RTK regulation, endocytosed EGFR can be subject to direct dephosphorylation by protein tyrosine phosphatases (PTPs) ([50] for review), including the endoplasmic reticulum (ER)-localized PTP1B which interacts with endocytosed EGFR at membrane contact sites between the ER and endosomes [51] (Box 1). The type 1γ phosphatidylinositol phosphate kinase variant i5 (PIPK1γi5) that phosphorylates phosphatidylinositol-4-phosphate to produce phosphatidylinositol-4,5-bisphosphate (PI4,5P_2_), was recently shown, together with its binding partner SNX5 (sorting nexin 5), to play a role in regulating the sorting of activated receptor onto ILVs [52]. Phosphoinositides play central roles in CME at the plasma membrane 53, 54, but this study suggests additional functions for PI4,5P_2_ at the endosome. Loss of PIPK1γi5 or SNX5 blocked EGFR sorting onto ILVs owing to increased Hrs ubiquitination, previously shown to prevent Hrs–EGFR interaction, resulting in enhanced and prolonged signaling.

Box 1 **PTP1B: paradoxical roles in EGFR downregulation and tumorigenesis**

The protein tyrosine phosphatase, PTP1B, is tyrosine phosphorylated (Y66) by activated EGFR, resulting in a threefold increase in its catalytic activity [99]. Moreover, ER-localized PTP1B has also been shown to both interact with and dephosphorylate activated endocytosed EGFR [100]. How this ER-localized phosphatase might interact with activated EGFR on the endocytic pathway was resolved by the identification of membrane contact sites (MCSs), regions of close membrane apposition (<20 nm), between the ER and endosomes [51]. In addition to its role in EGFR dephosphorylation, PTP1B activity also promotes the formation of ILVs and the lysosomal targeting of activated EGFR, likely via its dephosphorylation of the ESCRT-0 components Hrs and STAM 51, 101. Thus PTP1B activity is able to modulate both endocytic cargo and machinery, with downregulation of RTK signaling and therefore suppression of tumor development being the predicted outcome of its activity.

Consistent with a tumor-suppressor role, genetic ablation of PTP1B in p53-null mice resulted in accelerated lymphomagenesis [102], and increased ligand-stimulated phosphorylation of the EGFR was observed in PTP1B-null mouse fibroblasts [103]. Surprisingly, however, PTP1B knockout mice do not develop tumors and, although EGF-stimulated EGFR phosphorylation is increased, cell proliferation is not [104]. Indeed, PTP1B loss was found to diminish Ras activity through increased p62Dok phosphorylation [105], suggesting a potentially oncogenic role for PTP1B. This was confirmed when PTP1B activity was shown to promote ErbB2-dependent mammary tumorigenesis 104, 106. Activation of c-Src by PTP1B-mediated dephosphorylation of the inhibitory Y530 site [107] could account for tumorigenic effects of PTP1B activity [104]. Src kinase can interact with and phosphorylate RTKs including EGFR, and regulate proliferation through the Erk/MAP kinase pathway [108]; overexpression or increased activation of Src is found in several cancers including breast and colon cancers and is frequently linked with high EGFR levels [109]. In addition, PTP1B has recently been shown to promote the progression of prostate cancer [110], adding further evidence of a role for PTP1B activity in tumorigenesis and making it an attractive target for cancer therapy. PTP1B plays a pivotal role in the regulation of insulin and leptin, and is a target for diabetes therapies; small-molecule inhibitors of PTP1B have been developed which could have potential application to cancer therapy. However, further understanding of the mechanism and regulation of PTP1B activity is necessary before it can be exploited for therapeutic benefit.

### Regulation of EGFR recycling

The importance of ubiquitination in targeting activated EGFRs for degradation is established; negligibly-ubiquitinated EGFR mutants that evade the degradative pathway were recently shown to be recycled to the plasma membrane [45]. Thus perhaps recycling might serve as a default pathway. However, the identification of several effectors of EGFR recycling suggests a more active and regulated process. Receptor recycling can occur either by the direct Rab4- and Rab35-regulated route to the plasma membrane or by a Rab11-dependent route via the perinuclear endocytic recycling compartment (ERC). The calcium-modulating cyclophilin ligand (CAML) was reported to associate with the activated EGFR kinase domain, promoting EGF-stimulated receptor recycling [55]. Recently Eps15S, a short isoform of Eps15, was identified that targets endocytosed EGFR for recycling through the Rab11-positive ERC [56]. In addition, the adaptor protein Odin, a target of Src kinase activity [57], was found to promote EGF-stimulated receptor recycling [58]. Although increased recycling is predicted to prolong signaling, Odin has also been described as a negative regulator of EGFR signaling [59]. This apparent contradiction is yet to be explained but may involve downstream Odin-mediated suppression of signaling pathways.

Other factors affecting the passage of EGFR through the endocytic pathway include Hrs phosphorylation and AMSH-mediated deubiquitination of the receptor, and loss of Hrs phosphorylation and AMSH activity are associated with increased EGFR recycling [46]. The activating ligand can also direct EGFR traffic, depending on the pH-dependent stability of ligand–receptor interactions [60]. EGF binding, for example, remains stable at the reduced pH in endosomes, allowing targeting to the degradative pathway, whereas TGF-α dissociates at endosomal pH. The consequent deubiquitination upon dissociation allows the receptor to escape lysosomal targeting, and instead be recycled to the plasma membrane, consistent with the increased mitogenic effects of TGF-α [61]. Furthermore, the EGFR dimerization partners can also dictate its fate. As well as forming homodimers, EGFRs can form heterodimers with other EGFR family members. Whereas on EGF stimulation EGFR homodimers are sorted for degradation, heterodimers fail to recruit Cbl, thereby evading degradation and are instead recycled. For example, EGFR/ERBB2 heterodimers, that are overexpressed in many human tumors, are internalized at a slower rate, with increased recycling to the cell surface [62].

### Alternative fates for endocytosed EGFR

Sorting of activated EGFR for lysosomal degradation (and therefore attenuation of signaling) or recycling to the plasma membrane (associated with prolonged signaling) is fundamental to the regulation of EGFR signaling. However, alternative fates for activated EGFRs are emerging, including traffic to both the nucleus and the mitochondria. Nuclear EGFR, that is reported to depend on the nuclear localization sequence within the EGFR juxtamembrane region that interacts with importin-β, has been proposed to act as a transcriptional regulator and modulator of DNA repair through interaction with DNA-dependent protein kinase (DNA-PK). Proposed mechanisms of nuclear translocation are further detailed in Box 2 and reviewed in [63]. There is, however, a large pool of literature analyzing EGFR trafficking that does not report EGFR transport to the nucleus, and the exact mechanisms of nuclear translocation remain controversial. EGFR transport to the mitochondria, both constitutive and in response to a range of stimuli, has also been reported, although the role of CME is unclear, and both CME-dependent [64] and endocytosis-independent mechanisms [65] have been reported. Mitochondrial EGFR was found to directly phosphorylate cytochrome *c* oxidase subunit II (CoxII), involved in regulating apoptosis, in a c-Src-dependent manner, with reduced Cox activity and cellular ATP being reported [64], and a role in drug resistance has further been suggested [66]. However, the mechanism of translocation to the mitochondria and indeed the function of mitochondrial EGFR remain to be fully defined.

Box 2 **Potential mechanisms for nuclear translocation of endocytosed EGFR**

Potential mechanisms for the translocation of full-length EGFR to the nucleoplasm are beginning to emerge. The recovery of EGFR in ER fractions following prolonged EGF stimulation [111] was the first indication that a subset of EGFR might follow the retrograde pathway from the endosomes to the ER that is taken by some endocytosed toxins.

The retromer protein complex participates in the retrieval of proteins from the endosome to the Golgi, and binds phosphatidylinositol 3,5-bisphosphate (PI3,5P_2_), a lipid synthesized by PIKfyve. PIKfyve dysfunction impairs endosome–Golgi transport, and a role for this pathway in nuclear transport is supported by the inhibition of ligand-stimulated EGFR trafficking to the nucleus in human bladder cancer cells in which PIKfyve function is impaired [112]. The COP1 coat protein complex mediates retrograde transport through the Golgi and to the ER, and depletion of COP1 was also recently shown to inhibit EGF-stimulated transport of EGFR to the nucleus [113]. EGF-stimulated retrograde transport of EGFR to the Golgi and subsequent translocation to the nucleus were also reported to depend on a membrane fusion event driven by the SNARE protein syntaxin 6 and on dynein-dependent transport along microtubules [114].

A role for the Sec61 translocon, Sec61β, in EGFR translocation from the ER to the cytoplasm has been proposed, with nuclear translocation of EGF-stimulated EGFR being inhibited by depletion of Sec61β [111]. Whether Sec61β is also involved in EGFR nuclear translocation following ligand-independent stimulation is unknown, and how the EGFR is recognized by this machinery and the molecular detail of the retrotranslocation remain unclear.

Membrane extraction of EGFR at the ER implies that EGFR must pass through the cytosol before nuclear import. The hydrophobic transmembrane domain of the EGFR suggests the involvement of molecular chaperones, but how the retrotranslocated EGFR escapes proteasomal degradation has yet to be demonstrated. A way to avoid the conundrum presented by soluble EGFR in the cytoplasm is suggested by a recent study reporting the presence of Sec61 and EGFR on the inner nuclear membrane [115]. Entry into the nucleus has been reported to involve association of the nuclear localization sequence of EGFR with importins [116], proteins required for the import of macromolecules through the nuclear pore complex, but the mechanism regulating this transport remains unclear.

Thus, in addition to the comparatively well-studied recycling and degradative trafficking pathways of endocytosed EGFR, alternative fates for the EGFR, including translocation to the nucleus (Box 2) and mitochondria, have been reported, and further studies will be necessary to elucidate the nature and regulation of the transport mechanisms involved.

## EGFR trafficking and cancer

Abnormal expression and dysregulated intracellular trafficking of the EGFR family of RTKs play important and well-recognized roles in oncogenesis. Mutations of EGFR have been identified in several types of cancer 67, 68, 69, and the EGFR is the target for an expanding class of anticancer therapies (reviewed in [70]). Trafficking defects resulting in mislocalization and poor downregulation of the EGFR are associated with enhanced signaling [71], which can lead to the development of cancer [72]. Here we describe the different mechanisms by which modulations in EGFR trafficking and function can lead to oncogenesis or alter the outcome of antineoplastic therapies.

### EGFR oncogenic mutations: trafficking defects

Overexpression and particular oncogenic mutations of EGFR lead to spontaneous dimerization of the receptor, resulting in receptor activation [73]. Two main types of mutant EGFRs have been identified in tumorigenesis, both of which are constitutively active: truncated EGFR mutants and those harboring mutations in the kinase domain [74]. A range of constitutively-active oncogenic EGFR mutants found in non-small cell lung cancer (NSCLC) traffic into the ERC, allowing them to engage in a preferential interaction with Src, a crucial partner for EGFR-mediated oncogenesis [75]. Synergy between Src and EGFR also occurs because these two kinases traffic together, and their colocalization promotes EGFR-mediated signaling [76]. Interestingly, the NSCLC-associated EGFR mutants appear to be impaired in their interaction with Cbl, resulting in their defective ubiquitination and degradation, contributing to their prolonged signaling [77]. However, the most common EGFR variant in glioblastoma, EGFR type VIII, which harbors a deletion of 267 amino acids in the extracellular domain – leading to a receptor that is unable to bind ligand but is constitutively active [78] – is downregulated after Cbl-mediated ubiquitination [79].

### Role of oncogenes in the modulation of EGFR trafficking

Several oncogenes have been proposed to exert their action by affecting EGFR trafficking. One such oncogene, the Rho GTPase guanine nucleotide exchange factor Vav2, known to regulate cell adhesion, motility, spreading, and proliferation in response to growth factor signaling, has been shown to delay EGFR internalization and degradation, and enhance EGFR, ERK, and Akt phosphorylation [80].

Another oncogene, activated Cdc42-associated Kinase ACK1, is a non-RTK that can integrate signals from numerous interacting partners, including Cdc42 and EGFR [81]. ACK1 interacts with ubiquitinated EGFR to facilitate EGFR degradation, via a mechanism involving phosphorylation of the Arp2/3 regulatory protein, cortactin, potentially providing a link between Arp2/3-based actin dynamics and EGFR traffic towards degradation [82]. A single somatic mutation in ACK1 that abrogates ubiquitin binding can stabilize the EGFR at the plasma membrane [83], thereby prolonging mitogenic signaling after EGF stimulation and making some cancers resistant to EGFR kinase inhibitors such as gefitinib.

Another example of the importance of trafficking regulation in tumorigenesis is the interaction between the tumor suppressors PTEN (phosphatase and tensin homolog) and SPRY2 (sprouty homolog 2). Reduced SPRY2 expression causes hyperactivation of PI3K/AKT signaling in a PTEN-dependent manner, resulting in increased cell proliferation and invasion in prostate cancer [84]. The consequent positive feedback results in increased EGFR internalization and sustained signaling at the early endosome, in a mechanism involving activation of the stress-inducible p38 MAPK by PI3K. As previously described 85, 86, activated p38 facilitates clathrin-mediated EGFR internalization and evasion of degradation, and this allows the sustained signaling observed at early endosomes.

The examples above illustrate the conflicting role of EGFR traffic in signaling, with endocytosis resulting in either positive or negative effects on signaling and tumorigenesis, which is most likely due to complex interactions between EGFR and its downstream effectors.

### Anti-EGFR targeting drugs and combination therapies

Chemoradiotherapy, the combined treatment with two DNA-damaging agents, namely ionizing radiation and an alkylating agent such as cisplatin, is the standard choice of treatment for many cancers. Applying a combination of X-rays or chemotherapy and EGFR-targeting drugs of low general toxicity may enhance the lethal effect of local irradiation and/or revert tumor resistance [61]. To date, two different categories of compounds targeting EGFR have shown antitumor activity: monoclonal antibodies (mAbs; e.g., cetuximab) and low molecular weight tyrosine kinase inhibitors (TKIs; e.g., gefitinib), which target extracellular and intracellular domains of the receptor, respectively [87].

Gefitinib has previously been shown to efficiently suppress ligand-stimulated endocytosis of EGFR in some cell types but not in others [88]. Cetuximab was been found to be internalized as an antibody–receptor complex with EGFR even though binding of the antibody prevents stimulation of EGFR by endogenous ligands, leading to overall downregulation of EGFR expression [89]. The effects of EGFR-directed antineoplastic therapies on its intracellular trafficking in conjunction with receptor activity have however not been clearly established and require further investigation.

### Secondary effects of antineoplastic therapies on EGFR trafficking and activation

Another aspect of the regulation of EGFR trafficking that is likely to play a key role in cancer development and patient outcome is the effect of current cancer therapies on EGFR traffic. Both X-rays and chemotherapy treatments are capable of generating reactive oxygen species [90] that lead to the inactivation of redox-sensitive, cysteine-based protein tyrosine phosphatases [91]. As a result of the altered equilibrium between basal cellular kinase and phosphatase activities, EGFR becomes phosphorylated and the kinase activity of the receptor is stimulated in a ligand-independent manner [92]. The EGFR itself can also become directly activated via modification of cysteine residues located in its active site [93]. This activation is accompanied by receptor internalization and elicits endocytic trafficking/signaling events that have not been fully characterized but are either p38- 85, 86 or Src-dependent 94, 95, and clathrin- and AP2 adaptor-dependent [96].

One previous study shows that abrogating p38-dependent EGFR internalization reduces the efficacy of chemotherapy-induced cell death [86]. This suggests that EGFR-mediated survival signaling might occur primarily from the plasma membrane, and therefore ligand-independent EGFR internalization would enhance the cytotoxic effect of chemotherapy drugs such as cisplatin. However, in another study cisplatin was shown to induce both p38-dependent EGFR internalization and EGFR-dependent PKB/Akt activation, leading to cisplatin resistance [85]. The biological effects of this internalization therefore require further clarification to determine possible synergistic effects of chemoradiotherapy and EGFR-targeting drugs.

### Atypical EGFR trafficking in response to chemoradiotherapy

Following ionizing radiation treatment, the EGFR has been proposed to directly enter the nucleus and promote DNA-PK-dependent non-homologous end-joining double-strand break DNA repair 97, 98. As described above, the mechanisms involved in this translocation are controversial, and several molecular pathways have been postulated (Box 2). Moreover, whether the effect of EGFR on DNA repair is direct or indirect remains to be established because such a role could be exerted, without entering the nucleus, via signaling platforms such as those found on early endosomes [14].

In addition, mitochondrial translocation of EGFR has been reported in response to stress and RTK inhibitor treatments, where it could exert anti-apoptotic effects after chemotherapy-induced cell death, therefore contributing to drug resistance [66].

## Concluding remarks

**Figure 2:**
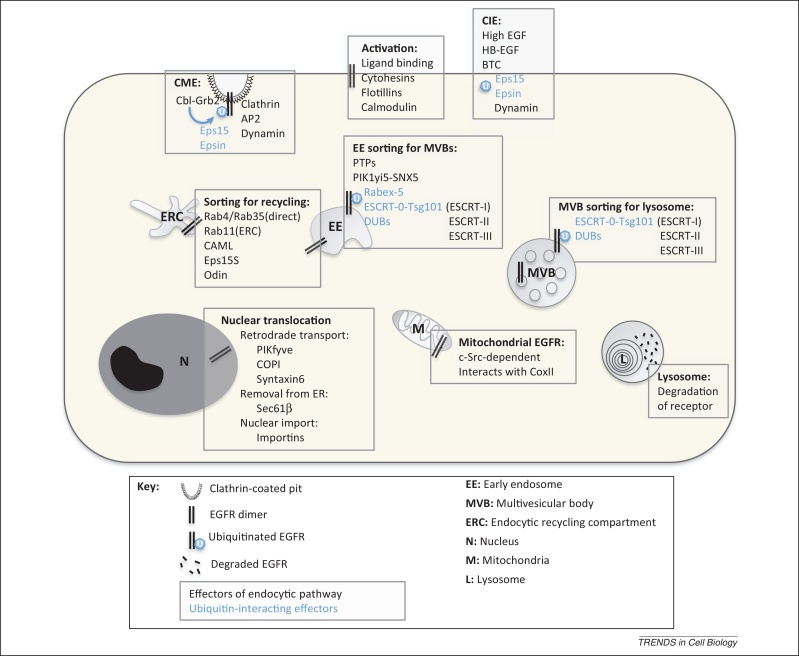
Effectors of ligand-stimulated EGF receptor (EGFR) trafficking pathways. Ligand binding mediates dimerization of EGFRs at the cell-surface, resulting in autophosphorylation, activation, and internalization. Under normal physiological conditions clathrin-mediated endocytosis (CME) is believed to be the major route of internalization, but clathrin-independent mechanisms have also been reported under conditions of potent activation [high concentrations of EGF or heparin-binding EGF-like growth factor (HB-EGF)/betacellulin (BTC)]. Internalized receptors are sorted at the endosome onto the recycling or degradative pathways, with ubiquitination targeting receptors for lysosomal degradation. Alternative fates reported for endocytosed EGFR, to the nucleus and the mitochondria, are also depicted.

Recent advances in our understanding of the molecular mechanisms regulating EGFR internalization, lysosomal targeting, and recycling (summarized in Figure 2) have increased our knowledge of the role that EGFR localization plays in the regulation of mitogenic signaling. This ongoing effort improves our awareness of factors underlying tumorigenesis and may also identify novel therapeutic targets. A comparatively new and exciting area is the additional role that EGFR activation and traffic may play in regulating responses to cancer therapy. Although there is growing evidence that EGFR signaling plays a role in cell survival and DNA repair in response to chemoradiotherapy, there is very little understanding of the role of internalization and post-endocytic traffic of EGFR in regulating that response. Determination of the trafficking pathways followed by stress-activated EGFR and the molecular mechanisms regulating that traffic will enable the analysis of the role of EGFR traffic in regulating DNA repair and cell survival. This will open up the prospect of development of future treatment strategies, including the targeting of factors involved in the regulation of cancer therapy-induced EGFR endocytosis and subsequent intracellular traffic. In combination with current drugs targeting the EGFR itself, controlling EGFR traffic could potentiate therapeutic action against tumors resistant to conventional chemoradiotherapy.
